# Further Observations on Strictures of the Rectum; with M. Boyer's Paper on Spasmodic Stricture of the Sphincter Ani

**Published:** 1822-09-01

**Authors:** 


*Further Observations on Strictures of the Rectum; with Remarks on the Opinions of some late Writers relative to the Situation of the Disease; and also, on Spasmodic Constriction of the Sphincter Ani; with a Translation of Part of M. Boyer's valuable Paper on that Complaint: accompanied with several Cases, and an Engraving.* By W. White, Member of the Royal College of Surgeons, London; Corresponding Member of the London Medical Society; and one of the Surgeons to the City Infirmary and Dispensary, Bath. Octavo, pp. 105. Bath, 1822.

In the Second Number of this series, for September, 1820, we gave an analysis of Mr. White's other work on the subject of anal diseases; it is now our duty to render some account of these "Further Observations" to our readers.

Our author commences by combating a pretty general idea, that strictures of the rectum are always the effect of inflammation. Extensive observation, he says, has convinced him that this, though sometimes, is comparatively rarely the cause of the disease. The length of time the disorder is known to exist—its limited nature, only occupying sometimes a very small portion in the circumference of the intestine—and the inner membrane having been frequently found in a healthy state on dissection, appear to him to be strong arguments against the doctrine of inflammation being always the cause of simple stricture. Extraneous bodies lodging in the rectum will often, of course, excite a diseased and contracted state of the gut. Our author next combats the general opinion, that strictures of the rectum rarely take place beyond the reach of the finger. On the contrary, it has so happened to him that, "in the course of an extensive practice, very few cases of the simple form of constriction have occurred so low down in the rectum, as to be within the reach of the finger." Mr. White is quite certain that the disease has been often overlooked, when the rectum has been subjected to an examination by the finger only. On looking over a list of 118 cases in his own practice, Mr. White does not find above six, where the stricture was within reach of the finger.

A permanent or spasmodic stricture in the rectum, or about the termination of the colon, presents often a great obstruction to the passage of the fæces, rendering the assistance of purgatives or injections indispensibly necessary. A distended state of the colon, as a consequence of stricture, claims the serious attention of practitioners, because it produces more distressing feelings to the patient, than he experiences at the stricture itself—a circumstance which is likely to mask the real disease, and lead to the suspicion of some affection of an abdominal viscus.

"Thus, for instance, should the distention be greatest at the superior portion of the ascending arch of the colon, where it lies under the liver, it may be mistaken for a disease of that organ, and this opinion will be further strengthened, should there be any obstruction to the passage of the bile through the common duct into the duodenum; which is not an unlikely circumstance to occur, from the pressure of the colon on that duct. If the distention be greatest in the course of its transverse arch, which passes under the stomach to the left hypochondrium, the functions of the stomach will be more or less disturbed by it, which will be particularly indicated by a great sense of fulness about the epigastric region soon after meals, especially if rather more than the ordinary quantity of food should be indulged in. Should there be an uneasiness, and sense of fulness about the left hypochondrium, where the colon descends before the spleen, previous to forming the sigmoid flexure, it may be mistaken for a disease of that organ.*" 12.

---

"* It is remarkable M. Boyer has also noticed, that spasmodic constriction of the sphincter ani has sometimes been mistaken for these and other complaints."

Repeated instances of inflammation of the colon from over-distention, have come within our author's knowledge, confirmatory of the observation of that excellent pathologist, Dr. Abercrombie. Mr. White coincides with M. Dupuytren, in opposition to several modern surgeons of this country, in believing that a permanent stricture of the sphincter ani may exist, independent of inflammation, fissure, or of constitutional or visceral disorder.

In the Journal Complementaire des Sciences Medicales, for November, 1818, there is an interesting paper by M. Boyer, one of the most distinguished surgeons in Paris, on a painful affection of the rectum. We had partly prepared an analysis of this paper, when we observed that Mr. White had given a translation of the greater part of it, we shall, therefore, take this opportunity of introducing the substance of it to our readers.

M. Boyer observes, that we may in vain search the ancients for a description of this disease, which he terms a flaw or fissure, (gerçure,) accompanied by spasmodic obstruction of the fundament. Lemonnier appears to have been the first who took notice of this disease, in the year 1689, in the following words:—

" ' These flaws, or fissures, are small painful ulcers, lancinating and without swelling—which follow longitudinally the wrinkles of the fundament, and which very much resemble those chaps or cracks which the cold produces on the lips and hands during winter—they are sometimes occasioned by the induration of the fæces, which becoming accumulated in a great quantity in the rectum, and afterwards evacuated, these through their excessive dryness and heat excoriate, or split the sphincter and the anus in passing away.'

" The author thinks that these fissures also may depend on dysentery, or venereal virus. He says they are superficial or deep, exterior or interior, tractable or malignant. In conclusion he proposes for their cure, the same means which are employed for other parts, namely, oils and fat combined with different vegetable and mineral substances." 19.

The species of fissure, however, which M. Boyer treats of, does not depend on any of the abovementioned causes—nor is it, he thinks, a disease of rare occurrence; having met with more than fifty cases of it in his own practice. Adults are almost exclusively the subjects of this complaint; he having never seen it in children or very young persons.

" ' No class of society appears to be exempt from it; both sexes are equally exposed to it; but women perhaps are more frequently attacked than men. The characteristic symptom of fissure is a fixed pain in one point in the circumference of the anus. This pain is always more acute during the alvine evacuations—it decreases, by little and little, between the intervals of evacuation. The sphincter of the anus is so contracted that the introduction of a finger, a candle, or a canula, is very difficult and excessively painful." 20.

The causes of this affection are obscure. It was generally found to have been preceded by hæmorrhoidal complaints.

"The complaint begins in an insensible manner: the dejection of the fæcal matter is accompanied with heat and smarting.—Some hours after the evacuation every troublesome sensation ceases; a patient thinks he has the piles, or that the parts are inflamed.—Sometimes these symptoms go off in the course of a few days, particularly if he abstains from heating drinks, uses clysters, and frequent ablution with cold water. But in a short time the heat and smarting re-appear; the expulsion of the fæces becomes more torturing, and the uneasiness it leaves lasts a longer time; the stools are a little tinged with blood; the pains increase; the laxative drinks to which we then usually resort, the clysters, and the cooling regimen afford a little relief. These means however cease to take effect; and in spite of their adoption, the disease continues its progress. Some patients are obliged to take a purgative medicine every forty-eight hours, and three or four clysters a day, to procure a stool; in other cases to use injection for hours together, till an evacuation takes place. If they remain many days without going to stool, the pains that they in the end experience in going, are still more excruciating —and they compare them to what a burning iron introduced into the rectum would produce. Some patients are then attacked with a sort of general convulsive catching, or fall into a swoon. There remains, after the evacuation, not only an acute pain, but prickings and throbbings, like those which are produced in an inflamed part. I have seen a woman in whom a febrile exacerbation succeeded every stool." 21.

The pains do not augment in an equal and progressive manner in this disease. They increase and diminish at intervals—often in consequence of certain circumstances, as violent exercise, vinous or spirituous potations, heating aliments, too much food. Indeed the influence of diet is so manifest in this complaint, that some patients have a horror of taking food. In some women, our author observes, the pains increased at the time of the menstrual discharge. In one case there was a regular hebdomadal exacerbation of the pain. Some patients cannot get ease from it without walking about.* He has known a man who, from this last cir-

* The writer of this article suffered from this complaint more than two years, and could not make out the nature of it till he read M. Boyer's paper. He could get no ease after a motion, until he had walked a couple of miles.

cumstance, was obliged to change his trade, and enter on a business where he had less sedentary employment.

" 'The pain which accompanies and that which follows the alvine excretion, is generally in proportion to the volume and hardness of the fæces. The more bulky contents are arrested by the constriction of the sphincter, and when they descend to the anus, they excite efforts excruciating, tedious, and useless, till they are softened by injections and the mucus secreted by the rectum. Even the evacuation of fæces, though of but little consistence, does not take place without pain. I have known a patient who experienced very acute pain, although he had a diarrhœa. Besides this, the passing of wind is also sometimes painful, difficult, or impossible. I cured a woman who, tormented with the desire, and (at the same time) impossibility of passing the wind collected in the intestines, was reduced to the painful inconvenience of keeping a probe, made of elastic gum, in the rectum.' " 23.

When the disease has lasted beyond a certain time, constitutional symptoms ensue, as emaciation, extreme nervous susceptibility, sometimes hypochondriasis—at others, retention of urine. The following were the appearances discovered by ocular and manual examination.

" 'Externally, nothing remarkable is to be seen. In some patients I have noticed hæmorrhoidal tumours; in others little pimples, which have always appeared to me, as well as the hæmorrhoids, to have no connexion with the fissure; in two or three only I have seen a slight discharge, which I believe equally foreign to that affection.

" 'In some cases, we may perceive in that point of the circumference of the anus where the patient feels pain, (it is commonly to the right or left,) we may perceive, I say, the lower extremity of the fissure; but in general we do not get a sight of it, without pressing on the opposite sides of the nates, and separating the orifice of the rectum a little: in some patients no endeavour will make it visible.

" 'The fore-finger does not penetrate into the rectum without difficulty; its introduction is always very painful; the pain is intolerable if we press forcibly on the fissure, and the patient throws himself forward to escape from the torment he suffers.

" 'The finger feels a remarkable constriction, tight, and continued: this constriction is one of the characteristic signs of the complaint. We perceive upon the mucous membrane of the intestine, no swelling nor hardness. Sometimes we remark, at a particular point, a depression elongated and parallel to the length of the intestine; at other times we only recognize the place which the fissure occupies, on account of the pain which the pressure we employ occasions on that part." 24.

This fissure, M. Boyer observes, is constantly accompanied by spasmodic constriction of the sphincters, although the constriction sometimes exists without fissure. The constriction with fissure is oftener observable than the constriction without fissure. Whether there be one, or both in conjunction, the symptoms are precisely the same, and they require the same treatment. M. Boyer believes the disease to be sometimes congenital—at least he has seen two persons in whom it commenced apparently with their existence. The fluidity and softness of the fœcal matter, in the early periods of life, render their expulsion easy, or more supportable; but in proportion as the patient advances in age, the alvine excretion becomes denser, and the pains of the anus more acute during and after each evacuation, which time renders progressively more difficult.

Among most of the patients who had been under M. Boyer's care, nothing but palliative means had been employed, and these often failed to give any relief.

" 'Among these means, some had for their aim the diminution of the consistence of the alvine contents; others, to allay the pain and heat of the fundament, and lessen its sensibility. Thus, they prescribed a cooling regimen, prohibited the use of stimulating diet and heating drinks. Some patients have, of their own accord, reduced their ordinary quantity of food to half, or even less; others have been compelled to the miserable plan of taking an aperient potion every two days. Most have made frequent use of simple or laxative enemas, and they have had recourse to them three or four times a day. These means at first procured some relief, but after a time they became useless, and scarcely produced a momentary alleviation. Fumigations of hot water; decoction of chervil or infusion of elder, cold effusion, general bathing, the hip-bath, the application of leeches, narcotic injections, suppositories, and opiate pastes, have sometimes rendered the pain more tolerable, but they have been always insufficient to the cure of the disorder, and often even to diminish suffering; however, I have once cured by some of these means a fissure of the anus, with slight constriction. The mode of treatment was long, and followed up with perseverance: I have obtained good effects from a pomade composed of hogslard, juice of house-leak, juice of night-shade, oil of sweet almonds—of each ℥iv.

" 'In most of these complaints which I have treated, I have employed these remedies before I proceeded to more powerful means.

" 'Many of these patients have made use of bougies to dilate the orifice of the rectum, but instead of diminishing the constriction, they have often had a contrary effect, the irritation caused by their pressure has sometimes increased the constriction of the sphincter to such a degree, that before long, the smallest candles, even a clyster-pipe could not overcome it; at other times without augmenting the constriction, the candles have so aggravated the pain, that the patients have not been able to bear it, have withdrawn them a few moments after they were introduced. In no case have I observed any good effects resulting from this plan, it has been always useless or pernicious." 29.

Baffled in general by all means of treatment, M. Boyer conceived a hope that the fissure might be remedied by converting it, by a simple incision, into a simple wound. He therefore operated, and his success exceeded his most sanguine expectations. The agonizing pains disappeared, and even the expulsion of the fæces over a raw surface was not by any means so painful as before. The fissure disappeared —the constriction ceased, and this result prompted him to try the same operation for spasmodic constriction without fissure, and the same success ensued.

" At a later period, having met with patients in whom the fissure occupied the anterior, or posterior part of the anus; parts on which a cutting instrumeat could not be used without inconvenience; I determined on making a latteral incision, without taking notice of the fissure which has always disappeared of itself after the operation.

" At last, experience has taught me that in a case of considerable constriction, one incision only is not sufficient, and that it is necessary to make two, one to the right and the other to the left, either at the same time or successively; either at a longer or shorter period as may be necessaray." 30.

As a preparative for the operation the patient takes, three days previously, a mild purgative, and on the day of the operation a laxative enema is thrown up. The steps of the operation we shall give in Mr. White's translation.

" 'I make the patient lie upon his side, as for the operation for fistula in ano; I carry the forefinger of my left-hand, anointed with cerate, into the rectum, and upon my finger I make a bistoury glide on its flat side, the blade of which is very narrow, square at the end, and the extremity rounded off. The edge of the bistoury is then directed towards the right or left side, according to the place which the fissure occupies, and with one incision I divide the intestinal membranes, the sphincters, the cellular tissue, and the integuments of the nates. I thus form a triangular wound, the top of which reaches to the intestine, and the base to the skin; it is sometimes necessary to elongate this, I do this with a second cut of the bistoury. In some cases the intestine slips away from the edge of the instrument, and the wound of the cellular tissue extends higher than that of the intestine; we must then introduce the bistoury a second time into the rectum to lengthen the incision of the intestine, or complete it with the blunt pointed scissars.

" 'When the constriction is great, I make two similar incisions, one to the right and the other to the left; and when the fissure is situated before or behind, I do not comprehend it in the incision.

" 'We introduce immediately into the wound, or the two wounds, a large bougie, which prevents the edges of the incised parts from reuniting in an irregular manner. We plug it up slightly with lint, apply a number of pretty long compresses, and the whole is supported by a bandage, like that which is used for fistula in ano. It is seldom that hæmorrhage supervenes, a slight compression is always sufficient to stop it. We do not remove the first dressing for three or four days, and afterwards dress it every day till the cicatrix is entirely formed, this is generally a month or six weeks, in some circumstances the cicatrization has not taken place till after the second month, or in the course of the third; but at other times, also, in twenty days—once only in fifteen.

" 'All the patients in whom I have performed this operation, have been cured radically, completely, and without return of the pain of the fissure, or the constriction." 32.

In Mr. White's experience, these spasmodic constrictions of the anus have been generally connected with stricture higher up the rectum. The great irritability of the sphincter will not permit the use of common hard bougies, but the soft bougie used by Mr. White has generally succeeded when employed with caution, and persevered in with courage and patience. Two cases, however, have lately occured to Mr. White, where the bougie failed, and he was obliged to have recourse to division of the sphincter.

For Mr. White's judicious observations on hæmorrhoidal and other tumours about the rectum—and also for a collection of interesting cases, some of them before published by himself and others, we must refer to the volume itself.

